# Differential lumbar spinal cord responses among wild type, CD4 knockout, and CD40 knockout mice in spinal nerve L5 transection-induced neuropathic pain

**DOI:** 10.1186/1744-8069-8-88

**Published:** 2012-12-18

**Authors:** Ling Cao, Holly Beaulac, Adriana Eurich

**Affiliations:** 1 Department of Biomedical Sciences, College of Osteopathic Medicine, University of New England, Biddeford, ME, USA

**Keywords:** Neuopathic pain, Nerve injury, CD4^+^ T cells, CD40, Microglia

## Abstract

**Background:**

Our previous studies have indicated that both lumbar spinal cord-infiltrating CD4^+^ T cells and microglial CD40 contribute to the maintenance of mechanical hypersensitivity in a murine model of neuropathic pain spinal nerve L5 transection (L5Tx). To further delineate the CD4 and CD40-mediated mechanisms involved in the development of L5Tx-induced neuropathic pain behaviors, we examined the lumbar spinal cord mononuclear cells of wild type (WT) BALB/c, BALB/c-CD4 knockout (KO), and BALB/c-CD40 KO mice via flow cytometry.

**Results:**

In WT mice, L5Tx induced significant but transient (at day 3 and/or day 7) increases of the total numbers of mononuclear cells, microglial cells (CD45^lo^CD11b^+^), and infiltrating leukocytes (CD45^hi^) in the ipsilateral side of the spinal cord. In CD4 KO mice, significant elevation of microglia was detected only on day 7 post-L5Tx, while no significant increase in infiltrating leukocytes post-L5Tx was observed. CD40 KO mice did not exhibit any of the changes observed in WT mice. Furthermore, neutralizing CD40 antibody treatment indicated an early involvement of CD40 signaling in the development of L5Tx-induced mechanical hypersensitivity.

**Conclusions:**

Altogether, data indicate that both CD4 and CD40 play a role in L5Tx-induced leukocyte infiltration into the lumbar spinal cord but have differential contributions to spinal cord microglial activation following peripheral nerve injury.

## Background

Neuropathic pain, defined as pain caused by a lesion or disease of the somatosensory system [1], is still largely treated suboptimally due to the limited understanding of the development and progression of neuropathic pain. Several research groups have demonstrated that central nervous system (CNS) glial cell activation and neuroinflammation are critical in the induction and maintenance of neuropathic pain in part through central sensitization [2-4].

Our laboratory has been investigating the underlying neuroimmune mechanisms in neuropathic pain using a murine model, spinal nerve L5 transection (L5Tx). Leukocyte infiltration into the affected nerve has been reported in human patients suffering neuropathic pain [5]. We are particularly interested in the roles of spinal cord infiltrating leukocytes and potential interaction between the infiltrating leukocytes and spinal cord resident glial cells. Previously, we have shown that L5Tx induced a significant but transient increase of CD4^+^ T lymphocyte infiltration into the lumbar spinal cord that peaked at day 7 post-L5Tx, and CD4 knockout (KO) mice displayed reduced mechanical hypersensitivity starting around 7 days post-L5Tx [6]. As recently reviewed by Grace *et al.*[7], pre-clinical studies have provided evidence suggesting the involvement of multi-level interactions among infiltrating leukocytes, particularly CD4^+^ T lymphocytes, microglia, astrocytes, and neurons along with resultant proinflammatory responses in the development of neuropathic pain. Both localized break down of the blood-spinal cord barrier following nerve injury [8] and production of chemokines within the spinal cord [9-11] are thought to be critical in facilitating leukocyte infiltration.

To further investigate the role of infiltrating CD4^+^ T lymphocytes in the maintenance of L5Tx-induced behavioral hypersensitivity, we proposed the potential involvement of the interaction between infiltrating CD4^+^ T lymphocytes and microglia via microglia-expressing CD40. CD40, a 48 kD cell surface tumor necrosis factor (TNF) family receptor, has been found to be upregulated in microglia upon activation both *in vitro* and *in vivo*[12-16]. CD40 has also been linked to the pathogenesis of various CNS diseases including multiple sclerosis and Alzheimer’s disease [17-19]. Our studies showed that both CD40 KO mice or bone marrow chimeric mice that have little CD40 expression in the CNS displayed significantly reduced L5Tx-induced mechanical hypersensitivity during the maintenance phase starting at 3–5 days post-L5Tx, suggesting the role of microglial CD40 in the development of L5Tx-induced mechanical hypersensitivity [16].

Our previous data indicate differential reductions of L5Tx-induced mechanical hypersensitivity when comparing CD4 KO and CD40 KO mice, that is CD40 KO mice displayed greater and earlier reduction of mechanical hypersensitivity [6,16]. We suspect the co-existence of distinct CD4- and CD40- mediated mechanisms in the development of neuropathic pain. Here, we further delineate these underlying mechanisms by examining lumbar spinal cord mononuclear cells collected from wide type (WT), CD4 KO and CD40 KO mice via flow cytometry. Follow-up studies with neutralizing CD40 antibody were performed to identify the specific timing of CD40 signaling involvement.

## Results

### Flow cytometric analysis of lumbar spinal cord mononuclear cells

**Figure 1:**
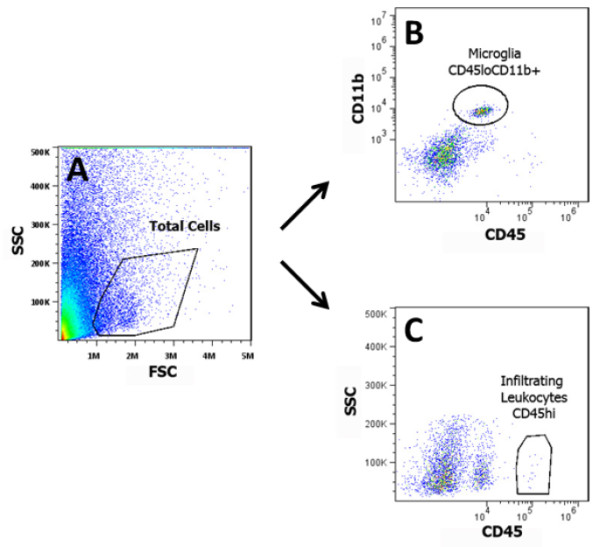
**Flow cytometric analysis of lumbar spinal cord mononuclear cells.** Total mononuclear cells collected from each sample (pooled ipsilateral or contralateral side of lumbar spinal cords from 3–4 mice) were labeled with mAbs against CD11b and CD45. All samples were examined with an Accuri C6 flow cytometer and analyzed with FlowJo. For each sample, total cell population was first identified (A), and then microglia (CD45^lo^CD11b^+^) (B) and infiltrating leukocytes (CD45^hi^) (C) were identified within the gated total cell population. The percentage of each of these populations within the total cells was recorded. The total number of microglia and infiltrating leukocytes per spinal cord were calculated based on the recorded percentages, the total mononuclear cells collected, and the number of mice used in each sample. Representative plots shown here are from a “WT day 3 L5Tx ipsilateral” spinal cord mononuclear cell sample.

**Figure 2:**
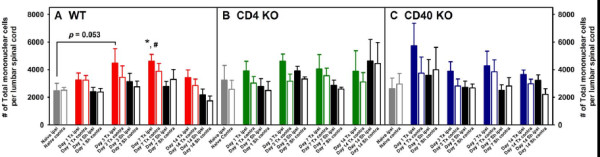
**Numbers of total mononuclear cells in lumbar spinal cord following L5Tx in WT, CD4KO, and CD40KO mice.** WT, CD4 KO, and CD40 KO mice were subjected to sham or L5Tx surgery. Lumbar spinal cord mononuclear cells from separate groups of mice of each genotype were collected at indicated times post-surgery. The temporal changes of the numbers of total lumbar spinal cord mononuclear cells in WT (A, n = 5–8), CD4 KO (B, n = 4) and CD40 KO (C, n = 4) mice are shown here (mean ± SEM). One-way ANOVA was performed to examine the basal level genotypic differences among naïve mice and no significant differences were found. Two-way ANOVA for data sets in each graph were performed. * indicates significant differences between the indicated group and all other groups at the same time point. ^#^ indicates significant differences between the indicated group and the corresponding Day 0 group. An additional significant result from statistical comparison is also shown within the graph. “Tx” = L5Tx, “Sh” = sham operation, “ipsi” = ipsilateral side, and “contra” = contralateral side. For naïve mice, “ipsi” = left and “contra” = right.

**Figure 3:**
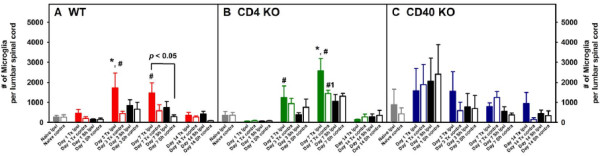
**Numbers of microglia (CD45**^**lo**^**CD11b**^**+**^**) in lumbar spinal cord following L5Tx in WT, CD4KO, and CD40KO mice.** Total mononuclear cells collected as described in Figure 2 were analyzed for their microglial content. The temporal changes of the numbers of lumbar spinal cord microglia in WT (A, n = 5–8), CD4 KO (B, n = 4) and CD40 KO (C, n = 4) mice are shown here (mean ± SEM). One-way ANOVA was performed to examine the basal level genotypic differences among naïve mice and no significant differences were found. Two-way ANOVA for data sets in each graph were performed. * indicates significant differences between the indicated group and all other groups at the same time point. ^#^ indicates significant differences between the indicated group and all other groups within the same treatment group (including the corresponding Day 0 group). ^#1^ indicates significant differences between the indicated group and days 0, 1 and 14 groups within the same treatment group. An additional significant result from statistical comparison is also shown within the graph. “Tx” = L5Tx, “Sh” = sham operation, “ipsi” = ipsilateral side, and “contra” = contralateral side. For naïve mice, “ipsi” = left and “contra” = right

**Figure 4:**
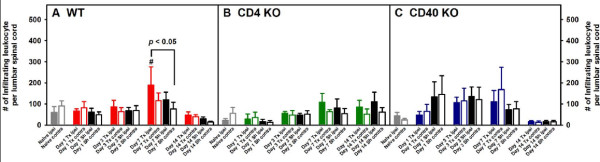
**Numbers of infiltrating leukocytes (CD45**^**hi**^**) in lumbar spinal cord following L5Tx in WT, CD4KO, and CD40KO mice.** Total mononuclear cells collected as described in Figure 2 were analyzed for their content of infiltrating leukocytes. The temporal changes of the numbers of lumbar spinal cord infiltrating leukocytes in WT (A, n = 5–8), CD4 KO (B, n = 4) and CD40 KO (C, n = 4) mice are shown here (mean ± SEM). One-way ANOVA was performed to examine the basal level genotypic differences among naïve mice and no significant differences were found. Two-way ANOVA for data sets in each graph were performed. ^#^ indicates significant differences between the indicated group and all other groups within the same treatment group (including the corresponding Day 0 group). An additional significant result from statistical comparison is also shown within the graph. “Tx” = L5Tx, “Sh” = sham operation, “ipsi” = ipsilateral side, and “contra” = contralateral side. For naïve mice, “ipsi” = left and “contra” = right.

Adult male and female WT, CD4 KO and CD40 KO mice were subjected to either L5Tx or sham surgery. At selected times, days 0 (naïve groups), 1, 3, 7, and 14 post-surgery, mice (for each genotype, 3–4 mice per treatment group) were euthanized and lumbar spinal cord samples (separated into ipsilateral and contralateral sides) were harvested and pooled to obtain mononuclear cells as described in the Materials and Methods (illustrated in Figure 1). Total mononuclear cells harvested from lumbar spinal cord are known to contain both infiltrating leukocytes and resident microglia. In WT mice, L5Tx induced a significant but transient increase of the total number of lumbar spinal cord mononuclear cells in the ipsilateral side of the spinal cord, which peaked between days 3 and 7 post-L5Tx (Figure 2A, two-way ANOVA, *p*_treatment_ = 0.003, *p*_time_ = 0.004, and *p*_treatment x time_ = 0.920). Although similar trends in the changes of the total lumbar spinal cord mononuclear cells were observed in both CD4 KO and CD40 KO mice, no statistically significant differences were detected in either type of KO mice (Figures 2B and C, two-way ANOVA, in B, *p*_treatment_ = 0.304, *p*_time_ = 0.392, and *p*_treatment x time_ = 0.968; in C, *p*_treatment_ = 0.131, *p*_time_ = 0.114, and *p*_treatment x time_ = 0.962). Further, total mononuclear cells were analyzed based on their microglial (CD45^lo^CD11b^+^) and infiltrating leukocyte (CD45^hi^) content (as illustrated in Figure 1). As expected, WT mice showed a significant increase of microglial number in the ipsilateral side on both days 3 and 7 post-L5Tx (Figure 3A, two-way ANOVA, *p*_treatment_ = 0.007, *p*_time_ < 0.001, and *p*_treatment x time_ = 0.460). Interestingly, significant increase of microglial number was only observed at day 7 post-L5Tx in CD4 KO mice (Figure 3B, two-way ANOVA, *p*_treatment_ = 0.091, *p*_time_ < 0.001, and *p*_treatment x time_ = 0.077), and no significant microglial increase was observed in CD40 KO mice at any time after L5Tx (Figure 3C, two-way ANOVA, *p*_treatment_ = 0.828, *p*_time_ = 0.348, and *p*_treatment x time_ = 0.955). Consistent with our previous report, significantly increased numbers of infiltrating leukocytes were detected in the ipsilateral side of lumbar spinal cord in WT mice at day 7 post-L5Tx (Figure 4A, two-way ANOVA, *p*_treatment_ = 0.422, *p*_time_ < 0.001, and *p*_treatment x time_ = 0.839). However, in either CD4 KO or CD40 KO mice, no significant increases of infiltrating leukocytes were observed within any treatment groups or sides relative to the surgery (Figure 4B and C, two-way ANOVA, in B, *p*_treatment_ = 0.824, *p*_time_ = 0.015, and *p*_treatment x time_ = 0.823; in C, *p*_treatment_ = 0.972, *p*_time_ < 0.001, and *p*_treatment x time_ = 0.988; for both B and C, no differences related to factor “time” were further identified within each surgery/side group with the SNK *post-hoc* test). In addition, there were no differences in all parameters measured among naïve mice at the basal levels across all three genotypes (Figures 2, 3 and 4, one-way ANOVA, *p* > 0.05).

### Effects of anti-CD40 treatment

**Figure 5:**
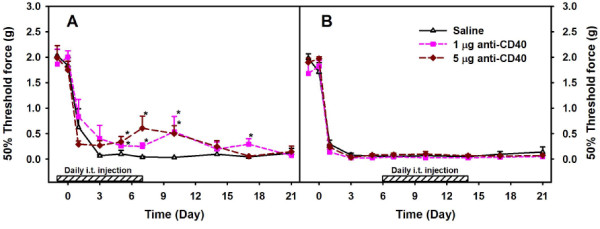
**Mechanical hypersensitivity of WT mice treated with anti-CD40.** WT mice were subjected to L5Tx. Neutralizing CD40 antibody was i.t. administered to WT mice daily either from day −1 to day 7 (A) or from day 6 to day 14 (B). Mechanical sensitivity of each mouse was tested before L5Tx and repeatedly after L5Tx using a series of von Frey filaments via the up-down method. All data are presented as mean ± SEM (n = 6). Two-way RM ANOVA was performed for data sets within each graph. * indicates the significant differences between the indicated group and the saline treated group within the same time point. In addition, within each treatment group, *p* < 0.05 between any time point post-L5Tx and both day 0 and day −1 time points (not indicated in the graph).

Our data from lumbar mononuclear cell analyses suggest that spinal cord CD40 might play a more important role compared to CD4 in the development of peripheral nerve injury-induced behavioral hypersensitivity in part through the inhibition of microglial activation and the infiltration of peripheral leukocytes into the spinal cord. We next examined the time when CD40 signaling is critical in the development of L5Tx-induced behavioral hypersensitivity. Neutralizing CD40 antibody was administered daily to WT mice either from day −1 to day 7 or from day 6 to day 14 via intrathecal (i.t.) injection (L5Tx was performed on day 0). Saline-injected mice were used as controls. As expected, mice who received saline (regardless of time period) displayed significant mechanical hypersensitivity after L5Tx that was maintained up to day 21 post-L5Tx (Figure 5) and is similar to what we have observed in non-treated WT mice following L5Tx surgery [6,16]. Further, when administered from day −1 to day 7, both doses of anti-CD40, 1 μg/day and 5 μg/day significantly reduced L5Tx-induced mechanical hypersensitivity (Figure 5A, two-way RM ANOVA, *p*_treatment_ = 0.0162, *p*_time_ < 0.0001, and *p*_treatment x time_ = 0.0096). However, this effect on mechanical hypersensitivity was gradually diminished following the last treatment of anti-CD40 and by day 21 post-L5Tx there was no difference in mechanical sensitivity among all treatment groups (Figure 5A). Interestingly, when administered from day 6 to day 14, anti-CD40 did not exert any effect on L5Tx-induced mechanical hypersensitivity (Figure 5B, two-way RM ANOVA, *p*_treatment_ = 0.132, *p*_time_ < 0.001, and *p*_treatment x time_ = 0.782).

## Discussion

Previously, we have shown that both infiltrating CD4^+^ T lymphocytes and spinal cord microglial CD40 are involved in the maintenance of L5Tx-induced mechanical hypersensitivity. To further identify the CD4- and CD40- mediated mechanisms, for the first time, we evaluated lumbar spinal cord mononuclear cells from WT, CD4 KO and CD40 KO mice via flow cytometry. It is known that both microglia and infiltrating leukocytes are important components within lumbar spinal cord mononuclear cells, thus we focused our investigation on these two populations.

First, our data showed that the total numbers of mononuclear cells collected from both CD4 KO and CD40 KO mice are comparable to that collected from WT mice at all selected time points post-surgery. Furthermore, for all parameters evaluated, there are no significant genotypic differences detected in naïve mice. These observations indicate that depletion of either CD4 or CD40 does not result in a reduction of total mononuclear cells within the lumbar spinal cord prior to any treatment, and differences detected among mice with different genotypes are most likely not due to the reduced availability of the specific cell populations that were analyzed.

In CD4 KO mice (that are known to have no CD4^+^ T cells), unlike in WT mice, no significant leukocyte infiltration was observed at day 7 post-L5Tx. This is consistent with our previous finding that CD4^+^ T cells are the dominant population detected at 7 days post-L5Tx in the lumbar spinal cord. Unexpectedly, our data indicate that a lack of CD4^+^ T cells can affect L5Tx-induced early increase of lumbar spinal cord microglial content. Increase in the total number of microglia is one of many signs of microglial activation during the development of neuropathic pain [2-4]. Although the peak time for detecting infiltrating CD4^+^ T cells is at 7 days post-L5Tx [16], it is possible that early-stage infiltrating CD4^+^ T cells play a critical role in microglial activation. In other words, lumbar spinal cord infiltrating CD4^+^ T cells potentially contribute to the maintenance of L5Tx-induced neuropathic pain in part through enhancing spinal cord microglial activation during the transition from the initiation phase to the maintenance phase of neuropathic pain behavioral development post-L5Tx.

In CD40 KO mice, neither increased leukocyte infiltration nor increased microglial number were observed following L5Tx as that in WT mice, indicating that CD40 is critical in both leukocyte infiltration and microglial activation post-L5Tx. Previously, we have demonstrated the role of CD40^+^ microglia in the maintenance of L5Tx-induced neuropathic pain behavior [16]. It has been shown that CD40-mediated, activated microglia are capable of releasing several chemokines, including CCL2 (also known as Monocyte chemoattractant protein-1 (MCP-1)), CCL5 (also known as regulated and normal T cell expressed and secreted (RANTES)), and CXCL10 (also known as interferon gamma-induced protein 10 (IP-10)) [20]. Together, we propose that depletion of CD40 significantly reduces the ability of spinal cord microglia to become fully activated, thus leading to a decreased production of various chemoattractant cytokines by activated microglia and subsequently reduced infiltration of peripheral leukocytes. Further, it is known that CD40 stimulation can lead to increased expression of certain adhesion molecules on both leukocytes and endothelial cells [21,22]. Elevated expression of adhesion molecules has been observed in activated microglia [13,23,24]. Thus, depletion of CD40 may limit an injury-induced increase of the total number of microglia at the injury site by reducing the migration of microglia towards the injury site. Reduced expression of adhesion molecules by endothelial cells could also contribute to the reduced infiltration of peripheral leukocytes. Nevertheless, whether the CD40 depletion-related low level of microglial activation is due to the lack of active CD40 signaling during the development of neuropathic pain or CD40 depletion-induced developmental defects of microglia requires further investigation. In addition, we did notice somewhat larger variations in all parameters we measured within CD40 KO mice, particularly changes between day 1 and day 7 post-surgery. Although it seems that surgery induced a greater increase of the total microglial number (particularly at day 1) in CD40 KO mice than those in WT and CD4 KO mice, no statistical significance was found. Future studies aiming to investigate the properties of microglia in CD40 KO mice in more detail may help to explain this observation.

Given the critical role of CD40, to further identify the times when active CD40 signaling is necessary in the development of neuropathic pain behavior post-L5Tx, we performed a series of studies with CD40-neutralizing antibody in WT mice. Although CD40 contributes mostly to the maintenance of L5Tx-induced neuropathic pain behavior [16], our data suggest that both early (during the initiation phase) and continued activation of CD40 signaling are essential in maintaining neuropathic pain behavior. In the current study, even with the highest dose of anti-CD40, we were unable to reverse L5Tx-induced mechanical hypersensitivity to a comparable level that has been reported in CD40 KO mice post-L5Tx by us previously [16]. This could be due to either the limited blocking ability of the neutralizing antibody used or that CD40-depletion related intrinsic developmental defects of microglia also contributes to the reduction of L5Tx-induced mechanical hypersensitivity in CD40 KO mice. As discussed above, future studies will be carried out to further characterize the microglial population from CD40 KO mice.

## Conclusions

Altogether, our data indicate that both CD4 and CD40 play a role in L5Tx-induced leukocyte infiltration into the lumbar spinal cord but have differential contributions to spinal cord microglial activation following peripheral nerve injury. Studies with CD40 neutralizing Ab suggest that CD40 is required early on in order to promote the maintenance of L5Tx-induced mechanical hypersensitivity.

## Materials and Methods

### Animals

Both male and female WT BALB/c mice (7–8 weeks old) were purchased from the National Cancer Institute (NCI, Frederick, MD) and were allowed to habituate to the institutional animal facility for at least one week before experimental use (8–9 weeks old). As previously described in detail [6,16], breeding pairs for BALB/c-CD4 KO mice and BALB/c-CD40 KO mice were originally obtained from Dr. William T. Lee of the Wadsworth Center, New York State Department of Health and the Jackson Laboratory (Bar Harbor, ME) respectively, and currently maintained at the University of New England (UNE) animal facility by breeding respective homozygous KO mice. All mice were group-housed (3–4 per cage) with food and water *ad libitum* and maintained on a 12-h light/dark cycle. Initially, experiments with just male or female WT mice were conducted. Since no significant differences in all parameters measured were observed and production of just male or female KO mice were limited, mixed male and female populations were used in all later experiments (including experiments with both WT and KO mice). The overall ratio between male and female mice within individual genotypes used in each set of experiments was around 1:1. The Institutional Animal Care and Use Committee (IACUC) at UNE approved all experimental procedures used in this study.

### L5Tx and spinal cord sample collection

WT, CD4 KO and CD40 KO mice were randomly selected into either sham or L5Tx groups. L5Tx and sham surgeries were conducted following previously published procedures [6]. We have shown that L5Tx-induced mechanical hypersensitivity can be observed up to 21 days post-surgery in WT BALB/c mice, while both CD4 KO and CD40 KO mice displayed reduced L5Tx-induced hypersensitivity with greater and earlier reduction detected in CD40 KO mice [6,16]. Our data also indicated that the peak time for detecting lumbar spinal cord infiltration of CD4^+^ T cells is day 7 post-L5Tx, while the optimal time for detecting lumbar spinal cord microglial activation is day 3–7 post-L5Tx. Thus, for each genotype, lumbar spinal cord samples (separated into ipsilateral and contralateral sides according to the side of injury) were collected at days 0 (naïve), 1, 3, 7 and 14 post-surgery following transcardiac perfusion as described previously [6,16]. Separate groups of mice were used at each time point. Since pooled samples were needed for flow cytometric analyses (see below Flow cytometry), each set of experiments with 3–4 mice per group produced only one data point for each group (n = 1). In order to perform statistical analyses, the same experiment was repeated several times (each time with 3–4 mice per group), i.e. the “n” shown in the results and figures indicates the number of repeated experiments, not individual animals.

### Flow cytometry

As previously described, mononuclear cells were prepared from pooled (3–4 mice) mouse lumbar spinal cord tissue (separated into ipsilateral and contralateral sides according to the side of nerve injury) using a discontinuous Percoll (Amersham, Piscataway, NJ) gradient and labeled for flow cytometric analysis [6]. Briefly, spinal cord tissue was first homogenized and filtered through a 70-μm cell strainer (BD Biosciences, San Diego, CA). Total cell suspension was then separated with 40% /70% Percoll gradients and mononuclear cells were collected from the layer that was below 40% and above 70% gradient. After washing with phosphate buffered saline, the total mononuclear cell number was determined using a hemacytometer with trypan blue (Sigma, St Louis, MO) before staining. All monoclonal antibodies (mAbs) were obtained from either BD Biosciences or eBioscience (San Diego, CA). Cell surface Fc receptors were blocked by anti-mouse-CD16/CD32 (clone 2.4G2) and then stained with fluorescent-labeled mAbs: APC-anti-mouse CD45 (clone 30-F11) and PE-anti-mouse CD11b (clone M1/70). All events from each sample were collected using an Accuri C6 flow cytometer (BD Biosciences - Accuri, Ann Arbor, MI) and further analyzed with FlowJo software (Tree Star, Inc., Ashland, OR). As previously published [6,16], mononuclear cells harvested from lumbar spinal cord are known to contain both infiltrating leukocytes and resident microglia. It has been established that the pan-leukocyte marker CD45 can be used to distinguish infiltrating leukocytes from the CNS resident, monocyte-derived microglia by flow cytometry, with CD45^hi^ representing infiltrating leukocytes and CD45^lo^ indicating microglia [25,26]. It is known that the majority of microglia express CD11b regardless of their activation status, thus it is generally accepted that cells expressing CD45^lo^CD11b^+^ are identified as microglia [27,28]. As illustrated in Figure 1, for each sample, following the identification of total cell population within the SSC vs. FSC plot, CD45^lo^CD11b^+^ cells were identified as microglia and CD45^hi^ cells were identified as infiltrating leukocytes (Figure 1). Non-stained cells were included in each run as controls. Data were reported as total number/lumbar spinal cord (ipsilateral or contralateral side).

### Anti-CD40 treatment and mechanical sensitivity test

WT BALB/c mice were randomly divided into two groups corresponding to the two treatment regimens: 1) treatment was administered daily from day −1 to day 7 post-L5Tx; 2) treatment was administered daily from day 6 to day 14 post-L5Tx. Within each regimen, three sub-groups were included: saline control (5μl per injection), anti-CD40 at 1 μg/5μl per injection, and anti-CD40 at 5 μg/5μl per injection. All mice were subjected to L5Tx at day 0. Neutralizing CD40 antibody was obtained from eBioscience (clone HM40-3) and diluted with sterile saline to a final concentration at either 1 mg/ml (for 5 μg per injection) or 0.2 mg/ml (for 1 μg per injection). Saline or anti-CD40 at either dose was administered to each individual mouse through intrathecal (i.t.) injection as previously described [29].

All mice were tested for their mechanical sensitivity using a series of von Frey filaments (Stoelting, Wood Dale, IL) as previously described [6,16,30]. Baseline tests were performed before L5Tx and follow-up tests were performed at days 1, 3, 5, 7, 10, 14, 17 and 21 post-L5Tx. The person performing the behavioral test was blinded to the treatment groups of individual animals.

### Statistical Analysis

All data were graphed with SigmaPlot 10.0 (Systat Software, Inc. San Jose, CA) and analyzed using SigmaStat 3.5 (Systat Software, Inc. San Jose, CA). One-way analyses of variance (ANOVA) were used to examine the basal level genotypic differences among naïve mice. Two-way ANOVA (for comparing the results obtained from flow cytometric analyses for each genotype of mice) or two-way repeated measures (RM) ANOVA (for comparing the results of behavioral tests) were performed to examine the effects of the two factors, time and treatment group, which was followed by the Student-Newman-Keuls (SNK) *Post hoc* test. All data are presented as mean ± SEM when applicable. *p* < 0.05 was considered statistically significant.

## Abbreviations

ANOVA: Analyses of variance; CNS: Central nervous system; IACUC: Institutional Animal Care and Use Committee; i.t.: Intrathecal; IP-10: Interferon gamma-induced protein 10; KO: Knockout; L5Tx: Spinal nerve L5 transection; MCP-1: Monocyte chemoattractant protein-1; mAbs: Monoclonal antibodies; NCI: National Cancer Institute; RANTES: Regulated and normal T cell expressed and secreted; RM: Repeated measures; SNK: Student-Newman-Keuls; TNF: Tumor necrosis factor; UNE: University of New England; WT: Wide type.

## Competing interest

The authors declare that they have no competing interests.

## Authors’ contributions

LC designed the study, carried out the experiment using anti-CD40 neutralizing antibody, performed data and statistical analyses, and drafted the manuscript. HB participated in the flow cytometric experiments, including most experiments with wild type mice and some experiments with knockout mice. AE participated in the flow cytometric experiments, including some experiments with wild type mice and most experiments with knockout mice. All authors read and approved the final manuscript.
